# Transcriptional analysis of the *recA *gene of *Streptococcus thermophilus*

**DOI:** 10.1186/1475-2859-5-29

**Published:** 2006-09-14

**Authors:** Gabriele Giliberti, Loredana Baccigalupi, Angelina Cordone, Ezio Ricca, Maurilio De Felice

**Affiliations:** 1 Dipartimento di Biologia Strutturale e Funzionale, Università Federico II, Napoli, Italy; 2 Dipartimento di Scienze e Tecnologie Biomediche, Università di Cagliari, Cittadella Universitaria, 09042 Monserrato, Cagliari, Italy

## Abstract

**Background:**

RecA is a highly conserved prokaryotic protein that not only plays several important roles connected to DNA metabolism but also affects the cell response to various stress conditions. While RecA is highly conserved, the mechanism of transcriptional regulation of its structural gene is less conserved. In *Escherichia coli *the LexA protein acts as a *recA *repressor and is able, in response to DNA damage, of RecA-promoted self-cleavage, thus allowing *recA *transcription. The LexA paradigm, although confirmed in a wide number of cases, is not universally valid. In some cases LexA does not control *recA *transcription while in other RecA-containing bacteria a LexA homologue is not present.

**Results:**

We have studied the *recA *transcriptional regulation in *S. thermophilus*, a bacterium that does not contain a LexA homologue. We have characterized the promoter region of the gene and observed that its expression is strongly induced by DNA damage. The analysis of deletion mutants and of translational gene fusions showed that a DNA region of 83 base pairs, containg the *recA *promoter and the transcriptional start site, is sufficient to ensure normal expression of the gene. Unlike LexA of *E. coli*, the factor controlling *recA *expression in *S. thermophilus *acts in a RecA-independent way since *recA *induction was observed in a strain carrying a *recA *null mutation.

**Conclusion:**

In *S. thermophilus*, as in many other bacteria,*recA *expression is strongly induced by DNA damage, however, in this organism expression of the gene is controlled by a factor different from those well characterized in other bacteria. A small DNA region extending from 62 base pairs upstream of the *recA *transcriptional start site to 21 base pairs downstream of it carries all the information needed for normal regulation of the *S. thermophilus recA *gene.

## Background

The bacterial RecA protein has an important role in a variety of cellular processes, such as the control of DNA status, repair of stalled replication forks, double-strand break repair, general recombination, induction of the SOS response and induction of temperate phages [1]. RecA has also been recently shown to possess other roles related to DNA metabolism, such as the apparent motor function in which DNA strand exchange is coupled to ATP hydrolysis [2]. In addition, roles of RecA in degradation of pectin in *Erwinia carotovora *[3], expression of adherence factors in *Vibrio cholerae *[4], pilus phase transition in *Neisseria gonorrhoeae *[5] and switching from pathogenic smooth to non-pathogenic rough cell form in *Pseudomonas tolaasii *[6], have been proposed and explained as secondary effects of RecA action on DNA structure and function.

However, RecA has been also associated to phenomena apparently not related to DNA metabolism, such as the adaptation of *Lactococcus lactis *to oxygen and heat shock [7,8] and of *Bacillus subtilis *to nutrient starvation [9]. Also in the moderately thermophilic lactic acid bacterium *Streptococcus thermophilus recA *expression is involved in the stress response mechanism [10]. *S. thermophilus *is a commercially important bacteria since it is used, along with *Lactobacillus *spp., as a starter culture for the manufacture of several fermented dairy foods. Its industrial use has substantially increased during the past two decades, as a result of the strong increase in consumption of dairy products. Such increase has led, as a consequence, to new demands on *S. thermophilus *performances, as stabile fermentation properties, consistent flavor and texture characteristics, resistance to bacteriophage infections. Research during the past two decades on the physiology of *S. thermophilus *has revealed important information on some of these properties, and more recently genome data have become publicly available [11]. Analysis of the *S. thermophilus *genome revealed a small size (1.8 Mb, probably the smallest genome of all lactic acid bacteria), a low G+C ratio (40%) and a phylogenetical relationship to mesophilic lactococci [12]. A *recA *insertional mutant of *S. thermophilus*, in addition to typical *recA *phenotypes (reduced growth rate and sensitivity to mitomycin C-induced DNA damages) also showed a strong reduction of viability and the appearance of a sub-population of morphologically altered cells in response to both heat shock and nutrient starvation [10]. These effects were independent from ClpL and GroEL homologues that were normally induced in the *recA *null mutant [10].

The transcriptional regulation of the *recA *gene has been studied in a variety of different bacteria. In *E. coli*, as well as in several other organisms, under physiological conditions *recA *transcription is repressed by the LexA protein that binds to its consensus binding site located in the promoter region of *recA *[13,14]. Upon DNA damage, RecA binds to single-stranded DNA regions generated by replication blocks, originating a nucleoprotein filament (RecA*) [15]. Activated RecA* possesses co-protease activity [15], required for self-cleavage of LexA [16]. The RecA*-promoted self-cleavage of LexA results in the inactivation of the repressor and thus in the induction of the SOS regulon including the *recA *gene [16]. In vitro experiments have shown that in the absence of RecA*, LexA is cleaved at high pH demonstrating that the protein is able to perform self-cleavage [17].

In the gram-positive model organism *Bacillus subtilis*, a LexA homologue is present and the *recA *gene is regulated with a mechanism similar to that studied in *E. coli*. The *E. coli *LexA and its *B. subtilis *homologue share a 52% similarity that becomes lower in the helix-turn-helix domain. As a consequence, the two proteins recognize different DNA target sites (5'-CTGTN_8_ACAG-3' for *E. coli *and 5'-CGAACN_4_GTTCG-3' for *B. subtilis*) [18].

In other bacteria different mechanisms of transcriptional regulation for the *recA *gene have been proposed. *Myxococcus xanthus *and *Deinococcus radiodurans*, although not similar to each other, both contain RecA and LexA homologues and control *recA *transcription with mechanisms that differ from the *E. coli *paradigm [19,20].

In *L. lactis *while a highly conserved RecA homologue is present [7] a LexA homologue has not been found. In this organism a different protein, HdiR, not homologous to LexA, has been shown to regulate several genes of the SOS system but not *recA *[8]. These evidence therefore suggest that while the RecA is highly conserved in prokaryotes, the mechanism of transcriptional regulation of its structural gene is less conserved.

We analyze here the expression of the *recA *gene of *S. thermophilus *and report evidence that *recA *expression and DNA damage-induction are exerted in a RecA-independent fashion through a factor not homologous to those well characterized in other bacteria.

## Results and discussion

### Mapping of the 5' terminus of the *recA *gene

**Figure 1:**
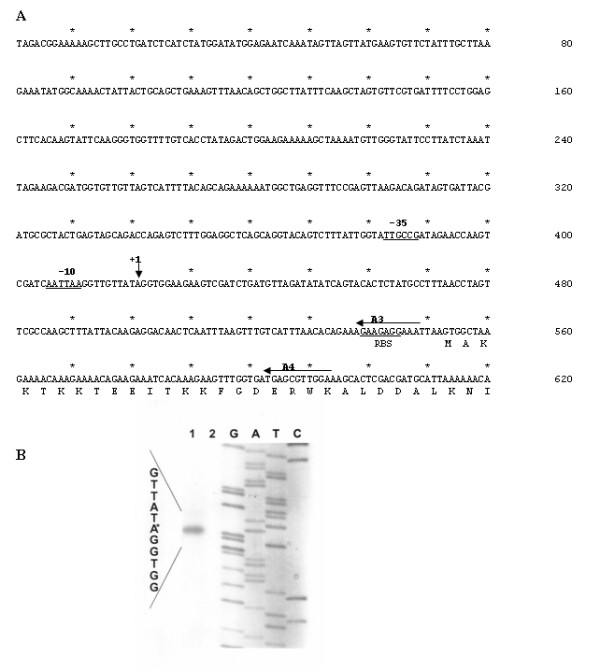
**The *recA *promoter region of *S. thermophilus***. (A) Nucleotide sequence of the *recA *promoter region of *S. thermophilus*. Deduced amino acid sequence of the 30 N-terminal residues of RecA is also reported. Horizontal and vertical arrows indicate synthetic oligonucleotide used in the primer extension analysis and the transcriptional start point. Regions of homology with the -10 and -35 consensus sequences are underlined. (B) Primer extension analysis performed with total RNA extracted from exponentially growing cells of *S. thermophilus *before (lane 2) and after (lane 1) exposure to sublethal concentration (20 ng/ml) of mitomycin C [10]. Primer extension and sequencing reactions were primed with the synthetic oligonucleotide A3. Similar results were obtained with oligonucleotide A4 (data not shown).

Primer extension experiments were carried out to map the 5' terminus of *recA *mRNA. Two radioactively labelled synthetic primers, designated A3 and A4 (Methods) and annealing the proximal part of *recA *mRNA (Fig. 1A), were used to hybridize *S. thermophilus *total RNA. Since it had been previously reported [3,21,22] that in several bacteria *recA *is expressed at a very low basal level and is transcriptionally induced by mitomycin C, we used total RNA from exponentially growing cells of *S. thermophilus *before and after exposure to sublethal concentration (20 ng/ml) of mitomycin C [10]. Reverse transcriptase was then used to generate cDNA primer extension products, which were separated by 6% polyacrilamide gel electrophoresis together with dideoxy sequencing ladders generated by using A3 and A4 primers and cloned *recA *DNA as a template. The results obtained with the A3 primer (Fig. 1B) were in agreement with the results obtained with the A4 primer (data not shown). The extension product obtained allowed us to localize the 5' terminus of *recA *mRNA 132 bp upstream of the beginning of the ORF. The extension product was found only when *recA *expression was induced with mitomycin C, suggesting that in *S. thermophilus recA *expression is strictly regulated. Sequences upstream of the 5' terminus (+1) resembled the conserved features of a typical promoter of an *E. coli *housekeeping gene, matching in three of six positions the consensus -10 and in four of six positions the consensus -35 (Fig. 1A).

### *recA *expression is induced by mitomycin C but not by heat shock or nutrient starvation

To analyze the expression of the *recA *gene we constructed a *recA::gusA *translational fusion. A 609 bp DNA fragment containing the *recA *promoter region and codons for 18 N-terminal amino acid residues of the *recA *ORF was amplified from *S. thermophilus *chromosomal DNA by using oligonucleotides P1 and P5 as primers (Methods). The PCR product was then fused in frame to the *gusA *gene of *E. coli *carried by plasmid pGU0, previously obtained by inserting the *gusA *coding region in the *Eco*RI site of the commercial plasmid pGemT-easy (Promega). The recombinant plasmid obtained, pGU1, was then used as a template for DNA sequencing reactions performed to verify that the *recA *and *gusA *genes were fused in frame (not shown).

**Figure 2:**
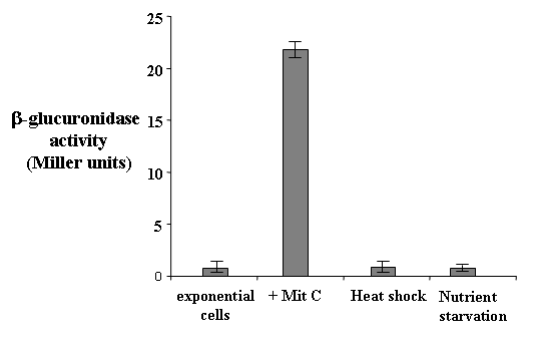
***recA*-driven β-glucuronidaseactivity**. Levels of β-glucuronidase activity in *S. thermophilus *strain Sfi39 containing the translational *recA::gusA*. Samples were harvested from exponentially growing cells, from exponential cells exposed to a sublethal concentration (200 ng/ml) of mitomycin C [10] or to heat-shock or nutrient starvation. Data are the average of three independent experiments.

The gene fusion was then transferred into the *E. coli – S. thermophilus *shuttle vector pNZ124, yielding plasmid pNG1 that was used to transform competent cells of the *S. thermophilus *strain Sfi39. The recombinant strain, S1, was grown at 42°C anaerobically in the presence and in the absence of a sublethal concentration (200 ng/ml) of mitomycin C [10] and exponentially growing cells collected by centrifugation and assayed for β-glucuronidase activity (Methods). Although the conditions of mitomycin induction differed substantially between the primer extension of Fig. 1B (20 ng/ml) and the β-glucuronidase assay of Fig. 2 (200 ng/ml) the two experiments together indicated that *recA *is poorly expressed in uninduced conditions and is strongly induced by mitomycin addition to the growth medium.

Since *recA *expression is involved in the cell response to heat shock and nutrient starvation [10], we decided to verify whether these stress conditions affected *recA *expression. Strain S1 was then grown at 42°C anaerobically to mid exponential phase and shifted at 50°C for three hours as previously reported [10]. A parallel culture was grown at 42°C for 48 hours to induce nutrient starvation, as previously reported [10]. Cell samples were collected and assayed for β-glucuronidase activity (Methods). In both stress conditions, the *recA*-driven β-glucuronidase activity was similar to that measured in exponentially growing cells not exposed to heat shock or nutrient starvation, thus indicating that both conditions do not affect *recA *expression (Fig. 2).

Results obtained with the primer extension experiment of Fig. 1 together with the analysis of the *recA*-driven β-glucuronidase activity of Fig. 2, suggest that expression of the *recA *gene is transcriptionally controlled and is inducible by DNA damages, caused in laboratory conditions by the presence of mitomycin C in the growth medium [10].

### Activation of *recA *expression is not RecA-dependent

A computer-assisted analysis of the recently released *S. thermophilus *genome [11] failed to reveal homologues of the *E. coli *or *Bacillus subtilis *LexA proteins (not shown). This analysis, however, identified in the *S. thermophilus *genome a homologue (YP_139374) of the HdiR protein that in *L. lactis *acts as transcriptional regulator of various SOS genes [8]. Although HdiR does not control the expression of *recA *in *L. lactis *[8], we searched for the HdiR putative consensus sequence (5'-tttATCAGtTtttCTGATaaa-3') [8] in the *recA *promoter region of *S. thermophilus*. Since no sequences matching the putative consensus for HdiR binding were found and since HdiR is not involved in *recA *regulation in the phylogenetically related *L. lactis*, it is likely that the protein controlling *recA *expression in *S. thermophilus *is not the HdiR homologue, YP_139374. Additional support for this conclusion comes also from the observation that HdiR acts in *L. lactis *in a RecA-dependent way [8] while the *S. thermophilus *factor is RecA-independent (see below).

**Figure 3:**
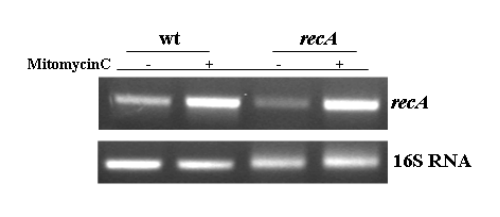
**Effects of RecA on *recA *transcription**. RT-PCR analysis performed on total RNA extracted from a wild type and a congenic strain containing a *recA *null mutation [10]. RNA was extracted from exponentially growing cells before or after exposure to sublethal concentration (20 ng/ml) of mitomycin C [10]. The 16S RNA gene was used as a standard to calibrate the amount of RNA to be used with synthetic oligonucleotides amplifying the *recA *gene.

Since in LexA-containing bacteria DNA damage-induction is RecA-promoted [15,16], we decided to verify whether in *S. thermophilus *such induction, although mediated by a different protein, was still under RecA control. To evaluate *recA *expression in a wild type and a *recA *null mutant we performed RT-PCR experiments with the synthetic primers, A4 and A5 (Methods). Total RNA was extracted from exponentially growing cells of the wild type and the *recA *null mutant strain before and after exposure to sublethal concentration (20 ng/ml) of mitomycin C [10] and measured spectrophotometrically. One-step reverse transcription-PCR (RT-PCR) were carried out by using a Access RT-PCR Kit (Promega). PCR products were analyzed by electrophoresis on a 2% agarose gel and the linearity of the reactions tested amplifying the 16S RNA gene by using various amounts of RNA for each sample as templates. Equal amounts of RNA were then used as templates in RT-PCR reactions to amplify the *recA *gene. As reported in Fig. 3, *recA *transcription was induced by sublethal concentration (20 ng/ml) of mitomycin C in both the wild type and the *recA *mutant strain. The induction conditions used for the experiment of Fig. 3 were identical to those used for the primer extension of Fig. 1B and the observation that a basal level of expression can be seen in Fig. 3 but not in Fig. 1B is most likely due to the higher sensitivity of the PCR-based approach.

These results indicate that the mitomycin-mediated induction of *recA *expression is controlled in *S. thermophilus *by a mechanism not requiring RecA, and thus different from those so far described in other bacteria.

### Minimal DNA region needed for *recA *repression and induction

**Figure 4:**
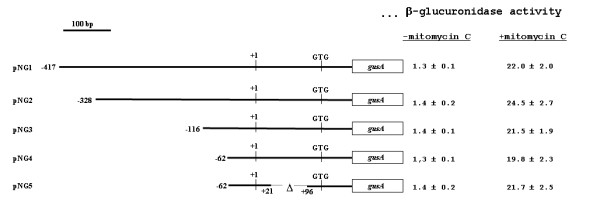
**Deletion analysis of the *recA *regulatoryregion**. The various plasmids carrying the *recA *promoter region translationally fused to the *gusA *gene and the β-glucuronidase activities obtained from cells containing those plasmids grown without and with sublethal concentration (200 ng/ml) of mitomycin C [10] are reported. For each plasmid transcriptional and translational start points are indicated. The extension of *S. thermophilus *DNA carried by each plasmid is indicated referring to the transcriptional start point as +1. Enzymatic data are the average of three independent experiments.

To study the mechanism controlling *recA *expression in *S. thermophilus *in more details we performed a deletion analysis of the *recA *promoter region. We started our analysis from plasmid pNG1, containing 417 bp upstream and 191 bp downstream of the *recA *transcriptional start site, and sufficient to ensure regulation and strong induction of the gene (Fig. 2). Similarly to what described for the construction of plasmid pNG1, a PCR-based strategy was followed to obtain plasmids in which the 609 bp insert of pNG1 was shortened at its 5' end of 89 (pNG2), 301 (pNG3) and 355 (pNG4) bp (Fig. 4). All plasmids were independently used to transform competent cells of the *S. thermophilus *strain Sfi39. Recombinant strains S1 (pNG1), S2 (pNG2), S3 (pNG3) and S4 (pNG4), were all grown anaerobically at 42°C and exponential cells collected and assayed for β-glucuronidase activity. As shown in Fig. 4, similar levels of β-glucuronidase activity were observed in all strains grown in the presence or in the absence of mitomycin C (200 ng/ml). These results therefore indicated that DNA carried by plasmid pNG4 contains all signals needed for the transcriptional regulation of the gene.

As determined with the primer extention of Fig. 1, the *S. thermophilus recA *gene has a 132 bp DNA region that is transcribed but not translated. To verify whether this region, present in plasmid pNG4, contains transcriptional signals we constructed plasmid pNG5 (Methods), carrying a 75 bp deletion within the 132 bp untranslated region (Fig. 4). Strain S5, carrying plasmid pNG5, was then assayed in parallel with strains S1, S2, S3 and S4 and showed similar levels of β-glucuronidase activity both in the absence and in the presence of the inducer mitomycin C (Fig. 4).

Our deletion analysis indicate that a DNA fragment of 83 bp, extending from 62 bp upstream and 21 bp downstream of the transcriptional start site, has all the information for regulation and full induction of the *recA *gene.

## Conclusion

1) We have characterized the *recA *promoter region of *S. thermophilus *and observed that expression of the gene is strictly regulated and induced by DNA damages.

2) Although RecA is required for *S. thermophilus *response to heat shock and nutrient starvation, expression of its structural gene is not affected by either stress condition.

3) Although functionally homologous to the LexA protein of other bacteria, the *S. thermophilus *protein controlling *recA *expression is not a structural homolog of LexA.

4) Unlike LexA of *E. coli*, the *S. thermophilus *protein controlling *recA *expression acts in a RecA-independent fashion.

5) An 83 bp DNA fragment containing the *recA *promoter and extending from 62 bp upstream to 21 bp downstream of the transcriptional start site, has all signals for regulation of the gene.

## Methods

### Bacterial strains, growth conditions and bacterial transformation

Strains used were *S. thermophilus *Sfi39 [23] and *E. coli *DH5α [24]. *S. thermophilus *was grown in anaerobic conditions in either HJL liquid medium or LM17 (lactose supplemented M17) solid medium [25]. The *E. coli *strain was grown aerobically in LB medium [24].

*S. thermophilus *and *E. coli *cells were transformed with plasmid DNA as previously described by electroporation [10] and by CaCl_2_-treatment [23], respectively.

### Primer extension analysis

Total RNA was extracted from exponentially growing cells before and after exposure to 20 ng/ml mitomycin C, by use of the RNeasy kit (QIAGEN). 50 μg of total RNA were used with γ^32^PdATP (NEN) labeled oligonucleotides (A3: 5'-CCTCTTCTTTCTGTG-3' and A4: 5'-CAACGCTCATCACCAA-3'), dNTP and AMV Reverse transcriptase (BRL) to prime cDNA synthesis, as previously described [26]. Reaction products were fractionated on 8M urea – 6% polyacrilamide gels alongside with DNA sequencing reactions primed with the same oligonucleotide.

### Reverse transcription-PCR analysis

Total RNA was extracted from a wild type and a isogenic strain carrying a *recA *null mutation before and after 30 min of exposure to 20 ng/ml of mitomicin C by use of RNeasy kit (Qiagen). Each RNA sample was treated with DNAse turbo (Ambion) following manufacturer instructions and the amount of RNA determined by spectophotometer. Identical amounts of RNA (200 ng) were then used in one-step RT-PCR experiments using ACCESS RT-PCR SYSTEM (Promega) and primer sets specific for the *recA *gene (A4: GGTGGAAGAAGTCGATCTGATG; A5 CCTTGCTCACCAGAATCAGGC) and for the 16S ribosomal gene (16S-for: CCGCAGCTAACGCATTAAGC; 16S-rev: GACTCGCAACTCGTTGTACC) used as RNA concentration control. PCRs were carried out with RNA alone to exclude that the amplification products could derive from contaminating genomic DNA.

### Plasmid construction

pGU0 plasmid was obtained by inserting a DNA fragment with *Eco*RI flanking ends and coding for the *gusA *gene of *E. coli *into the pGEMT-easy plasmid (PROMEGA) previously digested with the same restriction enzyme. A 609 bp DNA fragment, containing the *recA *promoter region (417 nucleotides upstream and 191 downstream transcriptional start site) was PCR amplified using *S. thermophilus *chromosomal DNA as a template and oligonucleotides P1 (5'-GCTTGCTGATCTCATCT-3') and P5 (5'-AAACCATGGCTCATCAT CACCAAACTTC-3') as primers. The PCR fragment was digested with the *Not*I restriction enzyme and cloned into pGU0, previously digested with the same enzyme, yielding plasmid pGU1.

The *recA::gusA *translational fusion was then moved by using *Sac*I and *SphI/NspI *restriction sites, in the pNZ124 vector, able to produce a RepA protein and, as a consequence, to replicate in *S. thermophilus*. The resulting plasmid, pNG1, was then used to transform competent cells of *S. thermophilus *strain Sfi39.

An identical strategy was followed to obtain plasmids pNG2, pNG3, pNG4 (containing respectively 328, 116 and 62 nucleotides upstream of the transcriptional start site) by pairing with primer P5 primers: P2 (5'-CTGCAGCTGAAAGTTTAACAGCTGG-3'), P3 (5'-GTGATTACGGAATTGCGCTTACTGGAGTAG-3') and P4 (5'-GCAGGTACAGTCTTTATTGG-3'), respectively.

Plasmid pNG5 was obtained by performing PCR reactions on pNG4 DNA as a template and oligonucleotides SMF1 (5'-AAACTCCCGGGTCAGATCGACTTCTTCCACC-3'; underlined is a *Sma*I site)-SM1 (5'-AAAATTTTCCAGCGCTACCGCTCG-3') and SMF2 (5'-TCTGACCCGGGAGTTTGTCATTTAACACAG-3'; underlined is a *Sma*I site)-SM2 (5'-CACCAACGCTGATCAATTCCACAG-3') as primer pairs. Amplified fragments were independently cloned into pGEM-Teasy vectors and the recombinant plasmids double digested with *Sma*I (inserted with oligonucleotides SMF1 and SMF2) and *Sca*I (present in pGEM-Teasy). Released DNA fragments of 1.564 and 2.090 bp were then ligated to produce an intermediate vector carrying the *recA *fragment of plasmid pNG4 with an internal deletion. This fragment was then used to replace the wild type *recA *sequence of pNG4 by double digestion with *Aor*51HI and *Ava*II restriction enzymes.

### β-glucuronidase assays

β-glucuronidase assays were performed as previously described [27]. For each sample a graph of *A*_405 _(Y-axis) versus time in minutes (X-axis) was designed; the slope *S *of the graph in *A*_405_units per minute was estimated and units of activity (nanomoles of *p*-nitrophenyl glucuronide hydrolysed per minute) were calculated from *S*/V_*e *_× 0.02; where V_*e *_is the volume of permeabilized cells in ml and 0.02 represents *A*_405 _given relative to 1 nmol of product produced. Specific activity equals units per *A*_590_. Values reported here were the average of at least three independent experiments. Statistical significance was determined by Student's *t *test and the significance level was set at *P *< 0.05.

## Competing interests

The author(s) declare that they have no competing interests.

## Authors' contributions

GG performed most of the experiments; LB performed the RT-PCR analysis of Fig. 3; AC contributed to plasmids construction and enzymatic assays; ER contributed to experiment design and discussion; MDF contributed discussions and suggestions during the work and helped in the preparation of the manuscript. All authors read and approved the final manuscript.
